# Quorum-Sensing Dysbiotic Shifts in the HIV-Infected Oral Metabiome

**DOI:** 10.1371/journal.pone.0123880

**Published:** 2015-04-17

**Authors:** Robert E. Brown, Mahmoud A. Ghannoum, Pranab K. Mukherjee, Patrick M. Gillevet, Masoumeh Sikaroodi

**Affiliations:** 1 School of Systems Biology, George Mason University, Prince William County, Fairfax, VA, United States of America; 2 Center for Medical Mycology, Case Western Reserve University, and University Hospitals Case Medical Center, Cleveland, OH, United States of America; 3 Microbiome Analysis Center, Department of Environmental Science & Policy, George Mason University, Prince William County, Fairfax, VA, United States of America; Ben-Gurion University of the Negev, ISRAEL

## Abstract

We implemented a Systems Biology approach using Correlation Difference Probability Network (CDPN) analysis to provide insights into the statistically significant functional differences between HIV-infected patients and uninfected individuals. The analysis correlates bacterial microbiome (“bacteriome”), fungal microbiome (“mycobiome”), and metabolome data to model the underlying biological processes comprising the Human Oral Metabiome. CDPN highlights the taxa-metabolite-taxa differences between the cohorts that frequently capture quorum-sensing modifications that reflect communication disruptions in the dysbiotic HIV cohort. The results also highlight the significant role of cyclic mono and dipeptides as quorum-sensing (QS) mediators between oral bacteria and fungal genus. The developed CDPN approach allowed us to model the interactions of taxa and key metabolites, and hypothesize their possible contribution to the etiology of Oral Candidiasis (OC).

## Introduction

Oral candidiasis (OC) is a major complication of HIV infection [1,2]. Clinical trials conducted by the OHARA and others showed that, although the incidence of OC has decreased in developed countries, it still is the most common oral complication in HIV patients and remains a significant complication in HIV-infected patients [3]. This study is focused on gaining insight into the interaction of *Candida* with other taxa in the oral metabiome, that should present hypothesis into the significant OC metabiome interactions, allowing for development of alternative preventative therapies.

Oral metabolites are products of the host, the oral bacterial microbiome (bacteriome), and the oral fungal microbiome (mycobiome). Functional shifts in the bacteriome and mycobiome may contribute to the difference in a healthy oral environment versus OC. Previously, we characterized the microbiome and mycobiome of a single cohort of healthy subjects [4] [5]. That research was extended to investigate the metabolites present in the oral cavity of the uninfected individuals (Controls, n = 12) versus the HIV-infected patients (Anti-Retroviral Therapy (ART)-experienced n = 8 and naïve n = 4). A comparison was performed on the control and HIV cohort metabolomic profiles to identify potential metabolites biomarkers for HIV. Our previous data analysis identified a significant (p<0.05) *differential* ratio of phenylalanine and tyrosine in response to HIV infection [6]. In this study there was no observable OC in n = 12 HIV participants.

The ART naïve sub-cohort (n = 4) is too small to perform significant stand-alone correlation analysis. However, the oral CD4 cell counts were comparable to the HIV ART (n = 8) cohort (see S1 Table). This indicates a similar systemic environment justifying the combination of the two HIV groups into one with n = 12. We note that by combining the two cohorts, we are increasing the statistical significance of those correlation contributed by HIV alone and also note that the ART cohort has enough samples to power the correlation analysis of those correlations contributed by ART. In summary, we are looking at the correlation differences between the Control cohort and all the correlation influenced by both ART and HIV alone.

Metabiome analysis, defined as the interrogation of the ecological relationships of bacteria, fungi, and host through metabolites and immune modulators, affords researchers the opportunity to analyze complex dynamic biological systems. The Correlation Difference Probability Network (CDPN), renamed from Differential Correlation Network (DCN) to emphasize it measures probabilities, is an analytical technique used to interpret the complex dynamics of the human metabiome, by identifying the significant correlation *differences* between disease and non-disease cohorts. By detecting biological pairwise feature differences, one has the opportunity to develop new hypotheses (Knowledge Discovery) that can then be experimentally tested.

We hypothesize that profiling the entire oral metabiome should lead to an improved understanding of how each component influences, or is impacted by, changes to the quorum-sensing relationships in the underlying disease. We anticipate these findings may form a basis for follow-on hypothesis and the development of experimental models to elucidate the influencers impacting the oral metabiome in HIV and use OC related pathways as the focus of the analysis.

## Results

### Multivariate Analysis

Oral rinse samples collected from uninfected individuals (n = 12) and HIV-infected patients (ART-experienced and—naïve, n = 12) were profiled and quantified for metabolites, bacterial genus, or fungal genus identification [4]. The resulting identified features that were measured in at least one of the 24 samples include: 198 host and microbial oral metabolites, 58 bacterial genera, and 39 fungal genera, with 28 additional unidentified metabolites [5].

### Correlation Analysis

**Fig 1:**
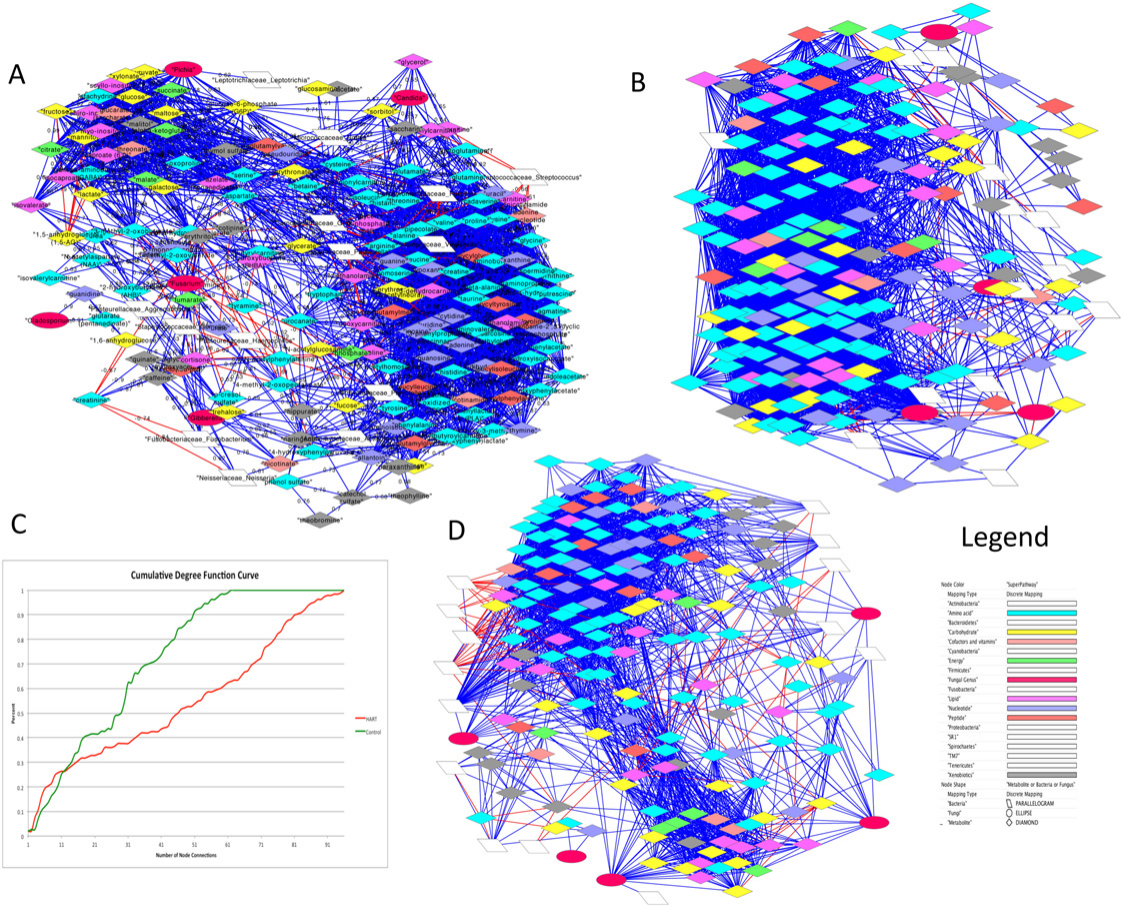
Correlation networks for Control and HIV cohorts. The legend is common for figures A, B and D: The correlation network represents features that are linked with a correlation coefficient greater than 0.6 (negative or positive) and with a p value <0.05. Parallelograms represent bacterial taxa, ellipses represent fungi and diamond shapes represent metabolites. Red edges represent a negative correlation between connected nodes and blue edges present positive correlations. A: Control correlation network with r. >0.6 or r.< -0.6 and p value <0.05. B: correlation network with +.0.6 < rho <-0.6 and p value <0.05. C: Cumulative Distribution Function D: Is the difference network map of 1A and 1B. It depicts those nodes that have a correlation with 0.6 < rho <-0.6 in either the control or HIV cohort, but not both.

We calculated Pearson correlations for the oral metabiome (metabolites, bacterial and fungal microbiome) that included 295 identifiable metabolites, fungi, and bacteria S2 Table. The analysis for the 295 features for both the Control cohort and the HIV cohort identified all significant correlations (rho >+0.6 or rho <-0.6). This subset of all correlations identified 2,681 Control correlations Fig 1A, and 4,240 HIV oral metabiome correlations Fig 1B. One fungus, *Pichia*, was only present in Control samples, and completely absent in the HIV samples.

The two network maps show a significantly different network complexity that is presented as a Cumulative Distribution Function graphing the cumulative distribution of the node frequency distributions of the control and HIV networks and shows a significant difference in network connectivity (Fig 1C, student t-test p = 0.0023). The network difference map shows correlations in the Control subset that are absent from the disease subset and vice versa and is presented in Fig 1D. The combination of relationships is still daunting to interpret as the difference mapping containing 2,658 non-redundant relationships. We narrowed the analysis to focus only on the correlations directly involving a bacteria, or fungus, with another metabolite, bacteria, or fungus (MBF network).

**Fig 2:**
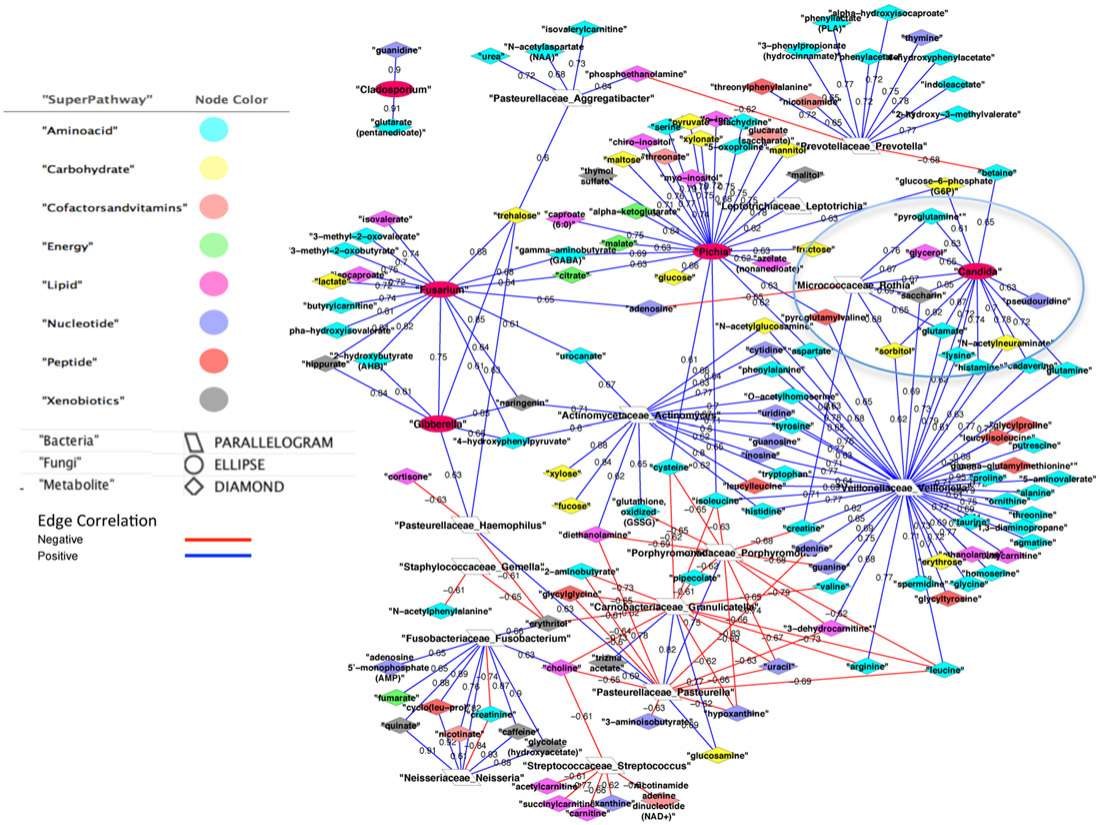
MBF Control cohort significant correlations (rho >0.6 or rho <-0.6). A blue edge connotes a positive correlation, red negative. The correlation value is listed on the edge. The node shape defines the type; diamond is metabolite, parallelogram is bacteria, and circle for fungi. The location of the node, and the link length, and orientation, are not important.

**Fig 3:**
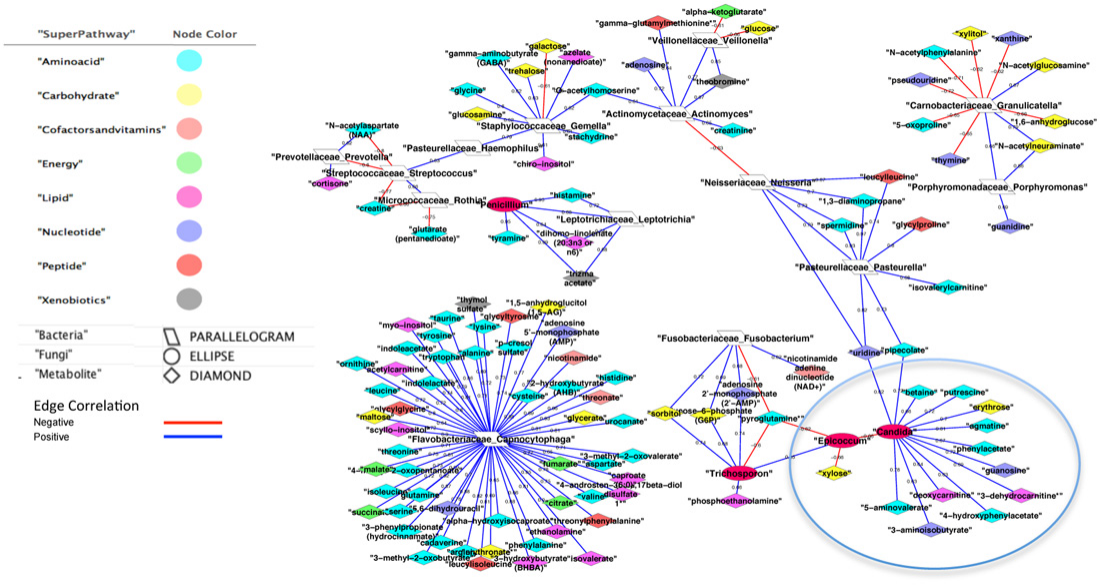
MBF HIV cohort significant correlations (rho >0.6 or rho <-0.6). A blue edge depicts a positive correlation; a red edge depicts a negative correlation. The correlation value is listed on the edge. The node shape defines the type; diamond is metabolite, parallelogram is bacteria, and circle for fungi. The location of the node, and the link length, and orientation, is not significant.

We present the significant correlations for the MBF Control cohort in Fig 2 and the MBF HIV disease cohort in Fig 3. Only 21 of the significant correlations with (rho <-0.6 or rho >0.6) in the MBF Control group overlapped with the MBF HIV significant correlation group. There were 452 significant Control correlations involving a bacteria or fungus with another feature of the possible 17,020 (0.8%). The significant oral metabiome correlations totaled 288 of the possible 17,205 (1.2%). S3 Table is the feature1 (node)—feature2 (node) pair listing with key metadata corresponding to correlation (edge) network relationships in the combined Figs 2 and 3.

In the Control cohort correlation network (circled in Fig 2) pyroglutamine, a cyclic monopeptide, was positively correlated with both *Micrococcaceae rothia* and *Candida* and has been implicated in quorum-sensing (QS) [7]. Additionally, *P*. *prevotella* was negatively correlated in the Control cohort with betaine, while betaine was positively correlated with *Candida*. Glycine betaine has been implicated in biofilm formation within microbial communities [8]. Additionally, the metabolite-taxa high correlations in the Control cohort include *Fusobacteriaceae fusobacterium* paired with the metabolite cyclo(leu-pro) a known antifungal compound produced by the bacterial genus *Lactobacillus*[9]. Other apparent biological significant correlations may be new areas for discovery, as in the positive correlations in the Control network in Fig 2. *Pichia’s* correlation with xylonate a metabolic partner of xylitol, and xylitol has been demonstrated as a quorum-sensing antagonist in gram-negative marker strain *Chromobacterium* [10].

Fig 3 represents the HIV cohort’s significant correlations, 1,3 diaminopropane that has been shown to be a quorum-sensing molecule for *P*. *pasteurella* [11]. 1,3 diaminopropane was also highly correlated with *Neisseriaceae neisseia* and could indicate communication between *Pasteurella* and *N*. *neisseia*.

*F*. *fusobacterium*, and the fungi *Epicoccum* and *Trichosporon* were all negatively correlated with Pyroglutamic acid. Pyroglutamic acid was a metabolic partner of pyroglutamine a cyclic monopeptide that is implicated in quorum-sensing, and is possibly indicative of communication between the two fungi and *F*. *fusobacterium* [7]. *Staphylococcaceae* correlates with acetylhomoserine, a known quorum-sensing molecule, and acetylhomoserine also was significantly associated with *Actinomycetaceae actinomyces* [12] [13]. Candida, a fungus, and the mold *Epicoccum* (circled in Fig 3) have non-metabolite mediated negative correlation relationship.

### Correlation Difference Probability Network (CDPN) Analysis

The Control correlation comprises either a bacteria, fungus, or metabolite (A_c)_ directly linked to another bacteria, fungus, or metabolite (B_c_). We denote the cohort correlation of A and B as A_h_-B_h_. Calculating the significant correlation difference probabilities (p<0.05) for the Control cohort A_c_-B_c_ versus HIV cohort A _h_-B_h_ resulted in 1,981 of the possible 17,020 (5.3%) being significantly different between the two oral environments. Of these 1,981 significant correlation differences, only 134 occurrences are direct taxa-taxa or taxa-metabolite difference relationships that may highlight biologically relevant oral metabiome changes.

**Fig 4:**
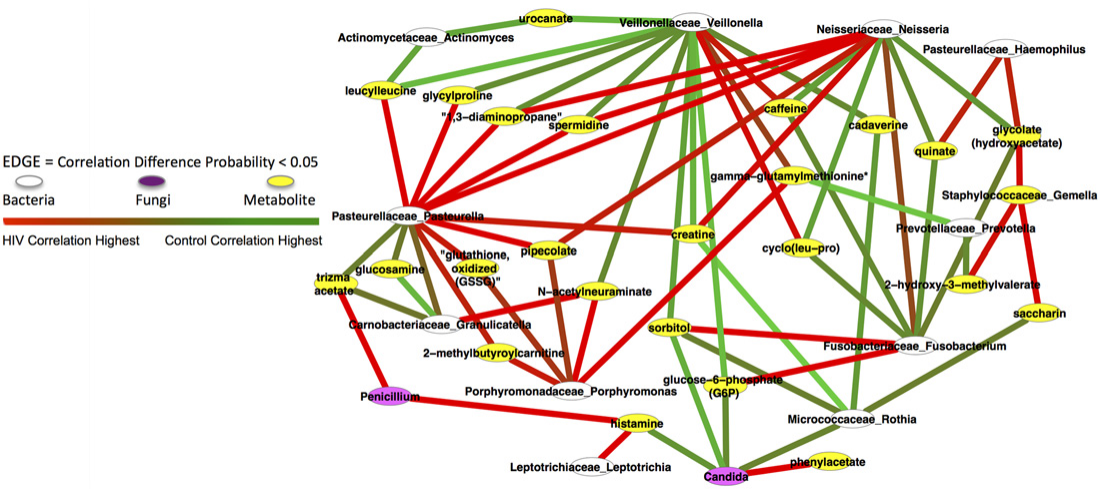
The Correlation Difference Probability Network (CDPN). Represents the significant Correlation Difference Probability Network for all Control vs. HIV cohort feature pair correlations (p<0.05). Fig 4 is the entire network. The position of node and edge length or not significant. Green edges indicate the Control Correlation was positive and the HIV cohort correlation was negative or very weakly positive. The red edges indicate the HIV cohort correlation was positive and the Control cohort correlation was negative or very weakly positive.

The Correlation Difference Probability Network (CDPN) in Fig 4 represents the significant difference network of Control and HIV correlations between pairs of features. The edge (link) color indicates the HIV correlation value for pair A-B was significantly different p<0.05, versus the correlation from the same Control A-B relationship. Typically, the significant difference probability pairing involves one cohort with a usually relatively large positive, or a rare large negative correlation, and the other cohort having only a weak positive, negative, or no correlation. S4 Table is the CDPN feature pair listing corresponding to Fig 4 and supporting raw data is in S2 Table.

One cluster of interest in Fig 4 is the subset of relationships involving Candida. Phenylacetate, a metabolite, is the only metabolite or species that had a significantly increased correlation with Candida. Decreased correlation differences with Candida were observed for histamine, sorbitol, and glucose 6 phosphate. Decreased significant correlation differences were observed for *Candida* and *M*. *rothia*. They are correlated higher (rho = 0.68,) in the Control group versus no correlation in the HIV group (p<0.01) implicating a possible inhibitory role of *M*. *rothia* allowing Candida to proliferate in its absence.

Beyond Candida, the CDPN enhances the recognition of intra and inter-species metabolites that are quorum-sensing or communication pathways across species. An example, *F*. *fusobacterium* paired with the metabolite cyclo(leu-pro) a known antifungal compound produced by the bacterial genus *Lactobacillus* [9]. Additionally, *Staphylococcaceae gemella* correlates with acetylhomoserine, a known quorum-sensing molecule, and acetylhomoserine also was significantly associated with *A*. *actinomyces* [12] [13]. We also see species-species significant difference correlation interactions, *N*. *neisseria* was more correlated with *Pasteurellaceae pasteurella* in the HIV cohort correlation (0.66), as was *Streptococcaceae streptococcus* and *M*. *rothia* with a correlation of 0.79. It was possible that this represent either multiple sub-significant intermediate correlations between the two taxa forcing the correlation, some undetermined metabolite or external factor not captured, or cell surface interactions between the species.

## Discussion

This study provided insight into variations between Control and the HIV oral environments that may explain the frequent expression of oral candidiasis (OC) caused by the unchecked growth of *Candida*, in a subset of subjects. We previously experimentally determined the metabolite, fungal, and bacterial components of the oral microbiome and shown that Pichia inhibits Candida biofilms, by modulating Candida growth [5]. Bacterial and fungal abundance ranges across the two cohort’s oral environments generally were shown to be of the same magnitude (S2 Table). We detected only six bacteria and fungi, **out of 97 fungi and bacteria genus** studied, where their abundance was significantly different (p<0.05) between the HIV and Control metabiomes.

The Correlation Difference Probability Network (CDPN) model of 198 metabolites, 58 genus of bacteria, and 39 genus of fungi shows promise as a platform for hypothesis generation. This study focused on only metabolite-taxa and taxa-taxa correlations. Notably, correlations do not define causation for the paired items but do imply a potential functional relationship. More frequently in the CDPN an inter-taxa metabolite intermediate indicates a quorum-sensing molecule as in *Staphyloccacaea gemella* and acetylhomoserine. Alternatively, the taxa sensing QS metabolite is altering its transcription pattern. Generally for positive correlations, this pattern would show a paired taxa(A)-metabolite-taxa(B) relationship where one taxa is initiating communication with the other. However, if the correlation pairing involves a negative correlation in one link in taxa(A)-metabolite-taxa(B) this may indicate either competition between the taxa or a virulence factor of one taxa impacting the other.

Direct taxa-taxa relationships (edges) may also be artifacts of an intermediary metabolite, related to both taxa, forming a triangular subnet, or may be indicative of surface proteins of one taxa enhancing or diminishing survivability of the other. The lack of *Pichia* in the HIV cohort likely contributes to the dysfunction allowing for OC [5]. We must be cognizant of the -0.6 < rho <+0.6 cutoff for correlations that reduces the visible single cohort correlation network’s size but should capture all direct significant correlation relationships in these small cohorts. However the correlation pair standard is not applied when determining correlation differences. All correlations are used from both cohorts since even a low correlation in one correlation can be part of a significant correlation difference when compared to a high correlation, positive or negative in the other cohort, and this scenario is generally the case.

With a CDPN taxa-metabolite pairing that is self-contained (no other taxa is associated with the metabolite), especially if the metabolite is a peptide or xenobiotic, may demonstrate an auto-inducing category of quorum-sensing. In Fig 3 we see the HIV cohort correlation of *S*. *gemella* and *A*. *actinomyces* with O-acetylhomoserine as an intermediary. O-acetylhomoserine is a member of Quorum-sensing (QS) molecules. It belongs to the class of *N*-acylated L-homoserine lactones (AHLs) and their cognate receptors (LuxR-type proteins) in Gram-negative bacteria in involved in mediating host-bacteria interactions [13].

There are high correlations in the Control cohort between *Candida* with sorbitol, histamine, and *M*. *rothia*. *M*. *rothia* or *Veillonellaceae veillonella* may be a source of sorbitol. We propose that when the sorbitol constraint is removed in the HIV cohort, *Candida* is allowed to proliferate with the reduced effectiveness of histamine co-immunologic polypeptides and metabolites, allowing the rapid *Candida* growth known as oral candidiasis. Sorbitol has been shown to cause osmotic stress on *Candida* that assists histidine associated polypeptide histidine 5 (Hst5) to extend beyond binding to the *Candida* cell wall allowing intracellular translocation to cause a cascade killing of the fungus [14]. The fungi *Penicillium* has a significant positive correlation with the metabolite histamine. That relationship suggests a cause and effect since it has been shown that *Penicillium* (a mold) triggers the release of histamine by the mucous membrane [15].

Enhanced *Candida* growth is supported by the significant correlation difference presence in the HIV cohort of PAA, a *Candida* biofilm metabolite of the phenylethyl alcohol catabolism pathway [16]. The quorum-sensing molecule phenylethyl alcohol may induce *Candida* to experience global up-regulation of central carbon metabolism and cell growth as a *Candida* biofilm auto-inducer. It has been shown that *Brasilense* supernatant has identified phenylacetic acid (PAA), an auxin-like molecule with antimicrobial activity [17]. It is possible in the HIV oral environment, *Candida* also presents PAA in certain circumstances that allows PAA’s antibacterial powers to reduce the abundance of bacterial species that normally keep *Candida* in check in the HIV Oral Microbiome, thus, reinforcing oral candidiasis to proliferate in the immuno-compromised individuals. From these relationships, we can generate hypothesis from this subset of the underlying network mechanism(s) that are altered to allow Candida to increase in abundance in the HIV cohort. If *M*. *rothia* is increased in the oral microbiome a corresponding decrease shall occur in Candidiasis. The reduction in histamine may be another factor that allows Candida to proliferate and via PAA reduce the inhibiting effect of oral bacteria on Candida growth.

One observation was that there are a number of interesting dipeptides and orphan xenobiotics that connect both bacterial and fungal genus in the CDPN. *Pasteurellaceae pasteurella* was linked to *V*. *veillonella* by glycylproline where there was a significant correlation difference between *P*. *pasteurella* and glycylproline in the HIV subjects while there was a decrease in the correlation difference between the glycylproline and *V*. *veillonella*. Even though there isn’t evidence in the literature that glycylproline acts as a quorum-sensing molecule it has the same core structure as many quorum-sensing molecules. This suggests that *P*. *pasteurella* was stimulated to produce glycylproline in the HIV subjects that inhibits the growth of *V*. *veillonella*, forming the basis of another hypothesis. There was a similar relationship between *P*. *pasteurella*- leucylleucine- *V*. *veillonella* and *P*. *pasteurella*- leucylleucine-*A*. *actinomyces*, again implying some form of communication.

More confounding is the direct significant correlation difference directly between *P*. *pasteurella* and *N*. *neisseria*, with a positive increase in this correlation difference in the HIV subjects. This latter relationship suggests that both genera are stimulated concurrently in the HIV subjects by an unknown factor.

Interestingly, there was a reverse relationship between Penicillium-trizma-*P*. *pasteurella* suggesting that *Penicillium* produces trizma in the HIV subjects that inhibits *P*. *pasteurella*. The observation that the orphan compound Trizma (TRIS), which is routinely used as a buffer in molecular biology, was present in the oral cavity is perplexing. It should be noted that TRIS was not used in processing of samples for the metabolome analysis, is not routinely used in toothpaste, and is present in the GC-MS spectra suggesting that it is a native compound in the oral cavity.

The finding of a significantly difference correlation between *Streptococcaceae streptococcus* and *M*. *rothia* (p<0.05) with the oral metabiome network model displaying a higher correlation in versus the Control is supported by the finding that these two bacteria combine also to form an oral biofilm [18].

Less frequently observed is a mid-level positive correlation in one cohort and another mid-level negative correlation in the other direction. This is the case with the *S*. *streptococcus* bacteria pairing directly with *P*. *pasteurella* significantly in the Control cohort (Fig 4). *P*. *pasteurella* and *S*. *streptococcus* have an intermediate positive Control correlation versus an intermediate negative correlation in HIV. In this case, neither the Control nor the HIV cohort has a particularly strong correlation, however, the correlation difference constitutes a very significant probability of p<0.01. This situation was one where both the disease and Control correlation values do not stand out significantly, but their difference correlations does. This DCN relationship may be indicative of the two bacteria being influenced by one or more other metabolites, host immune factors, bacteria, or fungi that individually are not significant, but appear so in combination. No dramatic shifts from high positive correlation to a very negative correlation in the other cohort were observed.

Additionally, we see numerous amino acids, lactones, intermediate metabolites, and xenobiotics embedded in the Correlation Difference Probability Network. Clearly, the numerous mono, di and tri amino acids derivatives, are very good candidates as quorum-sensing modulators. Other compounds like trizma acetate are orphans compounds with no obvious metabolic or environmental origin (this compound was detected in GC-MS so it was not introduced during sample preparation). Others, such as pipecolate, quinate, and urocanate could be as yet uncharacterized components of the oral metabiome quorum-sensing network.

We need to recognize this CDPN is only a partial representation of the entire oral environment. Other oral components, e.g. immune cells, play roles and are influencers supplying sources and sinks of metabolites, peptides, and other network determinants.

The DCPN of the oral metabiome, highlights cell signaling events that detect quorum-sensing and auto-inducer metabolites. These correlative changes do not *a priori* identify causality; however, they do elucidate underlying physiological process modifications in the disease state. The transformation between cohorts, from no correlation between a metabolite and taxa, to a significant correlation suggests quorum-sensing disruption, especially when the metabolite is also correlated to another taxa. Xenobiotic and/or cyclic peptides (lactones) are the most like QS molecules. If the taxa-metabolite is stand-alone that could be indicative of auto-induction. Therefore, a significant shift in correlations between disease and control samples, indicates an underlying metabolic change in the ecosystem.

Note that missing relationships, for example an intermediate metabolite linking two taxa, are possible with correlation differences analysis if the significant probability cutoff (p< 0.05) wasn’t achieved. Another issue is if one of the cohorts of the A-B pair lacks enough valid samples for probability determination (insufficient unique cohort sample values), the CDPN algorithm will be prevented from defining a correlation difference determination with one cohort correlation value undefined. This was the situation with Pichia having all immeasurable values in the HIV cohort; therefore no correlation could be established. Outlier values may have outsized influence over Pearson correlation results as well as too many immeasurable (below detection) data values.

In summary, we demonstrated that profiling the entire oral metabiome should lead to an improved understanding of how each component influences, or is impacted by, the underlying disease. We demonstrate the role of changing quorum-sensing molecule correlations with taxa represent underlying network modification in the disease state. These findings form a basis for follow-on hypothesis, and with increased cohort sizes in follow-up experiments, allow for the development of experimental models to elucidate the effects of treatments on the complex interconnected oral environment.

## Materials and Methods

This research was extended to investigate the metabolites present in the oral cavity of the uninfected individuals (Controls, n = 12) versus the HIV-infected patients (Anti-Retroviral Therapy (ART)-experienced and naïve, n = 12). The ART naïve sub-cohort (n = 4) is too small to perform significant stand-alone correlation analysis. However, the oral CD4 cell counts were comparable to the HIV ART (n = 8) cohort (see S1 Table). The raw data used for correlation and CDPN Sample analysis, abundances, correlations, and CDPN are in S2 Table.

This indicates a similar systemic environment justifying the combination of the two HIV groups into one with n = 12. There was an error in the data control sheet for sample 8; therefore, the bacterial analysis was not included for that sample.

We note that by combining the two cohorts, we are increasing the statistical significance of those correlation contributed by HIV alone and also note that the ART cohort has enough samples to power the correlation analysis of those correlations contributed by ART. The pipeline included the generation of intra-class Pearson correlation statistics for the Control and HIV cohorts and their corresponding -0.6> rho or rho >+0.6 magnitude correlation values, Figs 3 and 4. Abundances are used for bacteria and fungi, and quantities for metabolites. For simplicity only abundance will be mentioned, but both abundance and quantity are implied. Sample abundances A {a_1_, a_2_, a_n_} are paired with every other abundance B {b_1_, b_2_, b_n_}, where n = 12, creating metabiome pair abundance correlation values rho(A-B).

The differential correlation is the probability of there being a statistically significant difference (p<0.05) between both the Control cohort and HIV cohort pair A-B correlation values. The z-value statistical significance between the two sets of correlations is based on the individual cohort’s abundance pair correlations where C_1_ = rho_Control_(A-B) and C_2_ = rho_HIV_(A-B), and the actual total number of cohort sample values, N_1_ and N_2_, that were used to determine each metabiome pair’s correlation. Using Eq 1 the statistical significance z-value is determined [19].

$$z=\frac{1}{2}[\frac{log((1+C_{1})/(1-C_{1})-log((1+C_{2})/(1-C_{2})}{\sqrt{1/(N_{1}-3)+1/(N_{2}-3)}}]$$ Eq. 1

The significant differential correlation results create a Correlation Difference Probability Network map, Fig 4, requires a bacterium or fungi as at least one of the nodes in every edge pair, i.e. no metabolite-to-metabolite edges in the figure. Performing the CDPN analysis on the Control cohort abundances versus the HIV cohort abundances, we found the correlations were significantly higher in the Controls versus the HIV cohorts. The Figs 2, 3, and 4 are presented in Cytoscape [20].

## Supporting Information

S1 Table Summary of Participants Demographics. (DOCX)

S2 Table Oral HIV Study raw Metabolite quantities plus Bacterial and Fungal abundances. (XLSX)

S3 Table Metabolite Bacteria Fungi Control and HIV cohort significant correlations (rho >0.6 or rho <-0.6). (XLSX)

S4 Table Metabolite Bacteria Fungi Control CDPN Feature Pairs with a probability (p<0.05) difference between Control and HIV cohort correlations. (XLSX)

## References

[1] Shiboski CH, Wilson CM, Greenspan D, Hilton J, Greenspan JS, et al (2001) HIV-related oral manifestations among adolescents in a multicenter cohort study. J AdolescHealth 29: 109–114. 10.1016/s1054-139x(01)00280-4 11530311

[2] Shiboski CH (2002) HIV-related oral disease epidemiology among women: year 2000 update. Oral Dis 8 Suppl 2: 44–48. 12164659 10.1034/j.1601-0825.2002.00011.x

[3] Thompson GR 3rd, Patel PK, Kirkpatrick WR, Westbrook SD, Berg D, et al (2010) Oropharyngeal candidiasis in the era of antiretroviral therapy. Oral Surg Oral Med Oral Pathol Oral Radiol Endod 109: 488–495. 10.1016/j.tripleo.2009.11.026 20156694 PMC2843789

[4] Ghannoum MA, Jurevic RJ, Mukherjee PK, Cui F, Sikaroodi M, et al (2010) Characterization of the oral fungal microbiome (mycobiome) in healthy individuals. PLoS Pathog 6: e1000713 10.1371/journal.ppat.1000713 20072605 PMC2795202

[5] Mukherjee PK, Chandra J, Retuerto M, Sikaroodi M, Brown RE, et al (2014) Oral Mycobiome Analysis of HIV-Infected Patients: Identification of Pichia as an Antagonist of Opportunistic Fungi. PLoS Pathog 10: e1003996 10.1371/journal.ppat.1003996 24626467 PMC3953492

[6] Ghannoum M. A. M PK, Jurevic Richard R, Retuerto Mauricio, Brown Robert E., Sikaroodi Masoumeh, Webster-Cyriaque Jennifer, and Gillevet Patrick M. (2011) Metabolomics Reveals Differential Levels of Oral Metabolites in HIV-Infected Patients. PLoS Pathology. 10.1089/omi.2011.0035 PMC3545316 21751871

[7] Atkinson S, Williams P (2009) Quorum sensing and social networking in the microbial world. Journal of the Royal Society, Interface / the Royal Society 6: 959–978. 10.1098/rsif.2009.0203 19674996 PMC2827448

[8] Kapfhammer D, Karatan E, Pflughoeft KJ, Watnick PI (2005) Role for glycine betaine transport in Vibrio cholerae osmoadaptation and biofilm formation within microbial communities. Applied and Environmental Microbiology 71: 3840–3847. 16000796 10.1128/AEM.71.7.3840-3847.2005 PMC1169069

[9] Faust K, Sathirapongsasuti JF, Izard J, Segata N, Gevers D, et al (2012) Microbial co-occurrence relationships in the human microbiome. PLoS Comput Biol 8: e1002606 10.1371/journal.pcbi.1002606 22807668 PMC3395616

[10] Toivari M, Nygard Y, Kumpula EP, Vehkomaki ML, Bencina M, et al (2012) Metabolic engineering of Saccharomyces cerevisiae for bioconversion of D-xylose to D-xylonate. Metab Eng 14: 427–436. 10.1016/j.ymben.2012.03.002 22709678

[11] Henrik Christensen MFB, Anders Miki Bojesen, Magne Bisgaard (2011) Classification of Pasteurella sp. B as Pasteurella oralis sp. nov. ijsem. 10.1099/ijs.0.035246-0 21841008

[12] Yarwood JM, Bartels DJ, Volper EM, Greenberg EP (2004) Quorum sensing in Staphylococcus aureus biofilms. J Bacteriol 186: 1838–1850. 14996815 10.1128/JB.186.6.1838-1850.2004 PMC355980

[13] Campbell J, Lin Q, Geske GD, Blackwell HE (2009) New and unexpected insights into the modulation of LuxR-type quorum sensing by cyclic dipeptides. ACS chemical biology 4: 1051–1059. 10.1021/cb900165y 19928886 PMC2801563

[14] Jang WS, Bajwa JS, Sun JN, Edgerton M (2010) Salivary histatin 5 internalization by translocation, but not endocytosis, is required for fungicidal activity in Candida albicans. Mol Microbiol 77: 354–370. 10.1111/j.1365-2958.2010.07210.x 20487276 PMC2909388

[15] Meyer HW, Jensen KA, Nielsen KF, Kildeso J, Norn S, et al (2005) Double blind placebo controlled exposure to molds: exposure system and clinical results. Indoor Air 15 Suppl 10: 73–80. 15926947 10.1111/j.1600-0668.2005.00351.x

[16] Han TL, Tumanov S, Cannon RD, Villas-Boas SG (2013) Metabolic response of Candida albicans to phenylethyl alcohol under hyphae-inducing conditions. PLoS One 8: e71364 10.1371/journal.pone.0071364 23951145 PMC3741116

[17] Somers E, Ptacek D, Gysegom P, Srinivasan M, Vanderleyden J (2005) Azospirillum brasilense produces the auxin-like phenylacetic acid by using the key enzyme for indole-3-acetic acid biosynthesis. Applied and Environmental Microbiology 71: 1803–1810. 15812004 10.1128/AEM.71.4.1803-1810.2005 PMC1082559

[18] Filoche SK, Soma KJ, Sissons CH (2007) Caries-related plaque microcosm biofilms developed in microplates. Oral Microbiol Immunol 22: 73–79. 17311629 10.1111/j.1399-302X.2007.00323.x

[19] Morgenthal K, Weckwerth W, Steuer R (2006) Metabolomic networks in plants: Transitions from pattern recognition to biological interpretation. Biosystems 83: 108–117. 16303239 10.1016/j.biosystems.2005.05.017

[20] Shannon P, Markiel A, Ozier O, Baliga NS, Wang JT, et al (2003) Cytoscape: a software environment for integrated models of biomolecular interaction networks. Genome Res 13: 2498–2504. 14597658 10.1101/gr.1239303 PMC403769

