# Supplementary figures and images for: Quorum-Sensing Dysbiotic Shifts in the HIV-Infected Oral Metabiome
Source: PLoS One. 2015 Apr 17;10(4):e0123880. doi: 10.1371/journal.pone.0123880 (PMC4401692; doi:10.1371/journal.pone.0123880)

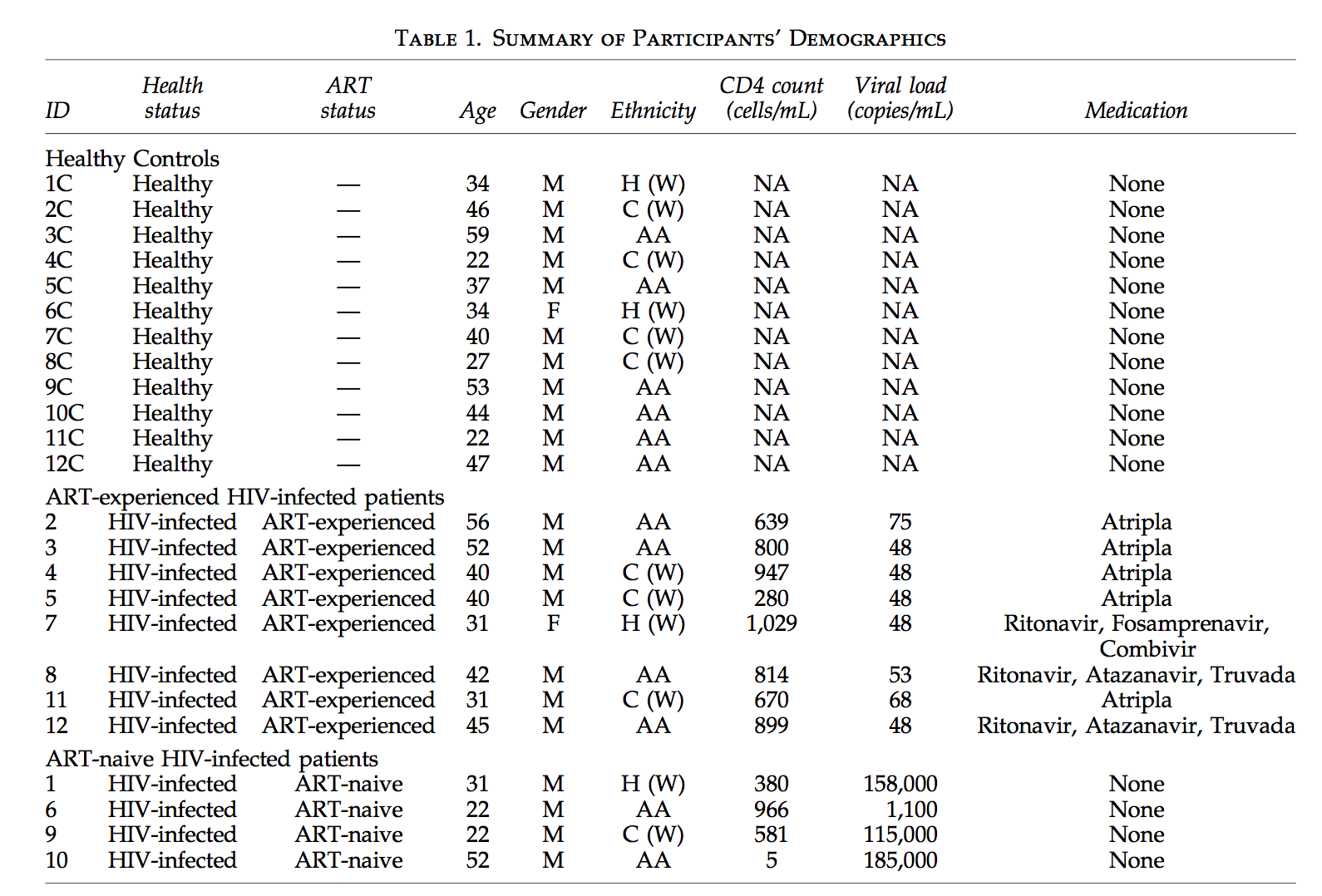

Supplement: S1 Table — (DOCX) [file pone.0123880.s001.docx]
